# Isolated Cataplexy in the Differential Diagnosis of Drop Attacks: A Case of Successful Clinical Diagnosis and Treatment

**DOI:** 10.1155/2012/757586

**Published:** 2012-07-17

**Authors:** Robert T. Egel, Anthony Lee, Thomas Bump, Alexander Javois

**Affiliations:** ^1^Division of Pediatric Neurology, Department of Neurology, Advocate Christ Medical Center and Hope Children's Hospital, 4440 West 95th Street, Oak Lawn, IL 60453, USA; ^2^Department of Neurology, and Adult Epilepsy Center, The University of Chicago, 5841 South Maryland Avenue, MC2030, Chicago, IL 60637, USA; ^3^Division of Cardiology, Department of Internal Medicine, Advocate Christ Medical Center, 4440 West 95th Street, Oak Lawn, IL 60453, USA; ^4^Department of Pediatrics, Division of Pediatric Cardiology, Advocate Christ Medical Center and Hope Children's Hospital, 4440 West 95th Street, Oak Lawn, IL 60453, USA

## Abstract

Drop attacks are sudden spontaneous falls that are not accompanied by alteration of consciousness and are followed by immediate recovery. Cataplexy, which is usually associated with narcolepsy, is one of the causes of drop attacks. We report a patient with the rare condition of cataplexy without associated narcolepsy (isolated cataplexy). Isolated cataplexy should be included in the differential diagnosis when a patient presents with recurrent drop attacks and normal diagnostic test results.

## 1. Introduction

Drop attacks (DAs) are spontaneous falls that are followed by quick recovery. In patients with syncope, DAs are associated with transient loss of consciousness. Causes of syncope include arrhythmias, aortic stenosis, orthostatic hypotension, neurally mediated syncope, subclavian steal, and other disorders. These conditions can also cause episodes of presyncope in which patients experience DA with lightheadedness rather than loss of consciousness. The evaluation of these patients includes history, physical examination, and 12-lead electrocardiography, supplemented when warranted by echocardiography, tilt table testing, stress testing, and event recordings.

 Falls may also result from seizures. If there is clinical suspicion of epilepsy, brain imaging and electroencephalogram (EEG) are useful diagnostic tools. Absence of epileptogenic activity during an attack suggests nonepileptic origin, but a normal interictal EEG does not rule out epilepsy. Also, patients with vertigo can be subject to falls. It is important to distinguish between sensations of lightheadedness and spinning (vertigo). The presence of vertigo is suggestive of a vestibulopathy or central nervous system process. 

Occasionally, recurrent falls occur without alteration of consciousness. In the majority (64%) of these cases, the etiology of DA is not clearly established [1]. Conversion reaction may simulate a drop attack and can be inferred by the exclusion of neurological disease, the exclusion of feigning, and determination of a psychological mechanism. A patient presenting with acute recurrent falls without alteration of consciousness and with unremarkable cardiac, neurological, and electrophysiological testing should prompt consideration of cataplexy and narcolepsy. 

The term cataplexy is derived from the Latin word *cataplessa* (to strike down with fear or the like) and Greek *kata-plexis* (down-stroke). Henneberg (1916) named cataplexy, while Gelineau named narcolepsy (1880) [2]. Cataplexy is most often associated with narcolepsy (excessive daytime sleepiness) as a major component of the narcolepsy tetrad that also includes sleep paralysis and hypnagogic hallucinations. The International Classification of Sleep Disorders-2 considers cataplexy as the hallmark symptom of the narcolepsy syndrome [3]. Excessive daytime sleepiness is usually (but not always) the first symptom to appear, while cataplexy may be delayed in onset 1–30 years. Isolated cataplexy without associated narcolepsy has been reported with genetic associations [4–6]. 

The following case describes pitfalls in the diagnosis and management of a patient with clinically defined isolated cataplexy.

## 2. A Case Report

An 18 year-old right-handed female presented for neurological evaluation 9 months after the onset of recurrent DA. Her initial attack occurred while at a mall with friends. She could not recall an inciting trigger. The attack consisted of sudden loss of tone in the face and bilateral lower extremities. She fell to the floor without alteration of consciousness and sustained abrasions to her knees. Subsequently, she reported occasional precipitation of attacks by emotional shock such as surprise, excitement, or laughter. The events would also rarely be associated with premonitory lightheadedness without visual, olfactory, or sensory auras. 

She was born full-term by caesarean section due to failure to progress and had normal birth weight. Her early development was unremarkable. At 2 years of age, she had varicella and also received varicella vaccine in college due inadequate blood titers. Immunizations were complete. There were no drug allergies.

Past medical history included left parietal closed head trauma sustained in a snowboarding accident at age 15 years, resulting in transient 24 hour amnesia and 1 week of decreased concentration without residual deficit. She had 2 clusters of chronic daily headaches of migraine without aura type that began less than a year before the onset of DA, lasted approximately 3 months per cluster and resolved without specific treatment. Her social history was negative for depression, psychosocial problems, or use of tobacco, alcohol, or illicit drugs. Her maternal aunt, paternal grandfather, and female maternal cousin have a history of migraine. A younger brother has attention deficit disorder and Tourette's syndrome. There is a family history of diabetes, but no history of narcolepsy or cataplexy. General physical and neurological exams were normal.

The initial emergency department evaluation revealed a normal complete blood count, comprehensive metabolic panel, urine pregnancy test, electrocardiogram (ECG), and computerized axial tomography of the head. She was referred to a cardiologist with a presumptive diagnosis of syncope. The cardiologic evaluation included lipid profile, ECG, 24-hour Holter recording, and 2D echocardiogram, which were normal. Neurally mediated syncope was diagnosed and she was advised to liberalize fluid intake, add salt to her diet, and not stand in one place for prolonged periods of time. 

Over the next month, she continued to have recurrent DA at a frequency of up to 12 episodes per day. The DAs were stereotypic, characterized by slumping of the torso and head with generalized loss of tone and no alteration of consciousness. Within 10 seconds after the event, she was capable of standing and resuming her activities. She did not report excessive daytime sleepiness, sleep paralysis, or hypnagogic hallucinations. In addition, there was no history for insomnia or parasomnia. A neurology consultation again led to a diagnosis of syncope following normal ECG, magnetic resonance scan with and without contrast, EEG, and lumbar puncture. Six months later, a second cardiologist diagnosed neurally mediated syncope after a normal ECG, 2D echocardiogram, 24-h Holter recording (with captured clinical drop attacks) and tilt table testing. Upon resuming care with her original cardiologist, she underwent extensive ambulatory monitoring of heart rate, heart rhythm, and blood pressure yielding normal results, even during recorded clinical drop attacks. She was then given independent trials of fludrocortisone and midodrine that failed to ameliorate DA. A third cardiology opinion eventuated in a normal EEG, ECG, and tilt table test. 

A second neurology consultation led to the clinical diagnosis of isolated cataplexy. Key determinants of the diagnosis included the absence of other narcolepsy tetrad symptoms, preservation of consciousness, lack of pre-syncopal symptoms, and absence of abnormalities on cardiovascular monitoring during recorded drop attacks. The narcolepsy genetic test for DQB1∗0602 and DQA1∗0102 gene mutation was negative. In order to prevent further injury, it was important to initiate treatment urgently to terminate events. She was placed on amitriptyline 25 mg each evening and through serial increases of medication, the attack frequency was reduced from up to 12 episodes per day to complete resolution of the attacks over a 1 year period. However, while on amitriptyline 25–50 mg, she developed a viral gastroenteritis presenting with nausea and vomiting, which precluded taking medication. Over several days of her illness, she had a cluster of brief breakthrough cataplectic attacks that subsided once she was able to tolerate medication. Amitriptyline was increased to 50 mg twice per day. 

Further diagnostic studies including human leukocyte antigen (HLA) typing, polysomnography (PSG), multiple sleep latency test (MSLT), and cerebral spinal fluid (CSF) orexin level testing were offered but not performed due to the patient's refusal and resolution of symptoms from empiric treatment. After having no cataplectic attacks in over 1 year, the medication was tapered and discontinued without recurrence.

## 3. Discussion 

This case illustrates the importance of detailed clinical history and broad differential diagnosis. This patient's quality of life was dramatically improved by the initiation of medical therapy that targeted cataplexy. She became more confident in her ability to perform activities without collapsing in public. Interestingly, her symptoms recurred during an episode of gastroenteritis with suboptimal medication absorption, and then resolved after the illness. We believe this case likely represents isolated cataplexy, though the lack of HLA typing, PSG, and MSLT in this case are important limitations in the characterization of her disorder. 

Cataplectic attacks typically are precipitated by emotions such as anger (90%), excitement (82%), surprise (61%), elation (59%), sexual intercourse (37%), and embarrassment (36%) [7]. Patients may report a brief sensation of lightheadedness during which they are able to brace themselves to prevent serious injury prior to acute muscle weakness. The weakness may affect the entire body or be limited to muscle groups such as jaw, neck, shoulders, or lower extremity muscles. The patient does not lose consciousness during brief (<1 minute) events in which the EEG typically shows normal wakefulness. Events that last >3 minutes may be associated with alteration of consciousness. In longer events (>20 minutes), rapid eye movement (REM) sleep may occur and can be confirmed on the EEG. The prevalence of cataplexy has been reported in up to 29% of young adults with associated excessive daytime sleepiness [8]. The prevalence of isolated cataplexy is unknown. Rare, familial cases of cataplexy with or without associated excessive daytime sleepiness or sleep paralysis have been described [4–6] and a case of isolated cataplexy of more than 40 years duration has been reported [9]. Some patients have a gradual disappearance of cataplexy with aging, especially in hereditary cases [4].

The diagnosis of narcolepsy can be confirmed with positive results on polysomnography, MSLT, and HLA typing. Narcolepsy is strongly associated with the HLA alleles DQB1∗0602 and DQA1∗0102. More than 90% of narcolepsy-cataplexy patients across all ethnic groups carry a specific allele of HLA DQB1∗0602, while this allele is present in 12%–38% of controls across many ethnic groups [10]. Therefore, while a negative result may be suggestive that the possibility of narcolepsy with associated cataplexy is <10%, a positive result is nonspecific. Neuropeptides (hypocretins/orexins) play an important role in the regulation and maintenance of wakefulness. Approximately 90–95% of patients with narcolepsy-cataplexy have absent CSF levels of hypocretin 1 (orexin A), while CSF hypocretin 1 levels in patients with narcolepsy without cataplexy are usually normal [11]. Findings suggest a loss of hypocretin secreting neurons, possibly due to autoimmune or neurodegenerative process.

Cataplexy may be considered a transition from wakefulness directly to an atonic state as seen in REM sleep, triggered by emotional stimulus. This theory is supported by therapeutic improvement with the use of medications that have REM suppressing action. Medications to treat cataplexy typically have norepinephrine and serotonin reuptake blocking properties (tricyclic antidepressants such as amitriptyline) or stimulate presynaptic release of norepinephrine (amphetamines) [7]. Fluoxetine and venlafaxine have also been utilized. In 2002, sodium oxybate, the sodium salt of *γ*-hydroxybuterate (GHB) and a metabolite of gamma amino butyric acid (GABA), was approved by the FDA for treatment of cataplexy in patients with narcolepsy. It is thought to reduce cataplectic attacks by binding specifically to GABA_B_ and GHB receptors, although its exact mechanism is unknown.

## 4. Conclusion

The approach to accurate diagnosis and treatment in a multispecialty setting requires a comprehensive history and broad differential diagnosis. A potential diagnosis of isolated cataplexy should be recognizable by clinical history. Once common conditions have been ruled out, less common conditions must be revisited with a detailed history and physical examination to decrease stress on patients and families undergoing diagnostic testing, provide cost-effective decisions, and treat the condition appropriately. Clinically, our patient exhibited features of isolated cataplexy. Supportive to the diagnosis were the lack of symptoms of the narcolepsy tetrad, lack of alteration of consciousness or pre-syncopal symptoms, and absence of abnormalities on cardiovascular monitoring during recorded drop attacks. Successful resolution of symptoms during empirical treatment with amitriptyline provided the patient with both immediate and long-term improvement in quality of life. Isolated cataplexy is a rare but important consideration in the differential diagnosis of a patient with drop attacks without alteration of consciousness. 
